# Analysis of EAWAG-BBD pathway prediction system for the identification of malathion degrading microbes

**DOI:** 10.6026/97320630013073

**Published:** 2017-03-31

**Authors:** Subramaniam Sivakumar, Palanivel Anitha, Balsubramanian Ramesh, Gopal Suresh

**Affiliations:** 1 Department of Biochemistry, Sri Sankara Arts and Science College, Enathur – 631 561;; 2 Department of Biotechnology, Sri Sankara Arts and Science College, Enathur – 631 561;; 3 Department of Microbiology, Sri Sankara Arts and Science College, Enathur – 631 561;

**Keywords:** Insecticide, Malathion, Biodegradation, pathway prediction, environmental toxicity

## Abstract

Insecticides are the toxic substances that are used to kill insects. The use of insecticides is believed to be one of the major factors behind the increase in agricultural productivity in the 20th century. The organophosphates are now the largest and most versatile class of insecticide used and Malathion is the predominant type utilized. The accumulation of Malathion in environment is the biggest threat to the environment because of its toxicity. Malathion is lethal to beneficial insects, snails, micro crustaceans, fish, birds, amphibians, and soil microorganisms. Chronic exposure of non-diabetic farmers to organophosphorus Malathion pesticides may induce insulin resistance, which might ultimately results in diabetes mellitus. Given the potential carcinogenic risk from the pesticides there is serious need to develop remediation processes to eliminate or minimize contamination in the environment. Biodegradation could be a reliable and cost effective technique for pesticide abatement. Since today as there were no metabolic pathway predicted for the degradation of organophosphates pesticide Malathion in KEGG database or in any of the other pathway databases. Thus in the present study, an attempt has been made to predict the microbial biodegradation pathway of Malathion using bioinformatics tools. The present study predicted the degradation pathway for Malathion. The present study also identifies, Streptomyces sp. and E.coli are capable of degrading Malathion through pathway prediction system.

## Background

The pathway is a series of consecutive enzymatic reactions that produce specific products. A Pathway can be defined as a modular unit of interacting molecules to fulfill a cellular function. A 2-D diagram depicting linkages of the protein and non-protein entities usually represent a pathway. A pathway is a representation of interconnected nodes (biomolecules) and modes (arrows). The pathway prediction system predicts plausible pathways for microbial degradation of chemical compounds. Predictions use biotransformation rules based on reactions found in the EAWAG-BBD database or in the scientific literature. PPS predictions are most accurate for the compounds that are similar to compounds whose biodegradation pathways are reported in the scientific literature; in environments exposed to air, in moist soil/or water, at moderate temperature and pH with no competing chemicals or toxins; and the sole source of energy, carbon, hydrogen or other essential element for the microbes in these environments, rather than present in trace amounts. In recent years, pathways prediction tools are gaining momentum, since this approach is proven to be fruitful in several biodegradation studies and reduced considerable money and time spent on wet lab research [1].

Insecticides are the toxic substances that are used to kill insects. Such substances are used primarily to control pests that infest cultivated plants or to eliminate disease-carrying insects in specific areas. The use of insecticides is believed to be one of the major factors behind the increase in agricultural productivity in the 20th century. Nearly all insecticides have the potential to significantly alter ecosystems; many are toxic to humans; and others are concentrated in the food chain.

The organophosphates are now the largest and most versatile class is parathion and Malathion; others are Diazinon, naled, methyl parathion, and dichlorvos. They are especially effective against sucking insects such as aphids and mites, which feed on plant juices. The chemicals’ absorption into the plant is achieved either by spraying the leaves or by applying solutions impregnated with the chemicals to the soil, so that intake occurs through the roots. The organophosphates usually have little residual action and are important, therefore, where residual tolerances limit the choice of insecticides. They bind to acetyl cholinesterase and other cholinesterases. This results in disruption of nerve impulses, killing the insect or interfering with its ability to carry on normal functions. Organophosphate insecticides and chemical warfare nerve agents (such as sarin, tabun, soman and VX) work in the same way. Organophosphates have an additive toxic effect to wildlife, so multiple exposures to the chemicals amplify the toxicity.

Malathion is an organophosphate parasympathomimetic, which binds irreversibly to cholinesterase. Malathion is an insecticide of relatively low human toxicity. In the former USSR it was known as carbophos, in New Zealand and Australia as maldison and in South Africa as mercaptothion. Malathion is one of the most widely used broad-spectrum insecticides used in the United States and throughout the world. It has been identified in atleast 21 of the 1,623 hazardous waste sites that have been proposed for inclusion on the EPA National priorities list (NPL). Malathion is a derivative of nerve gas received most of the attention in late 1999, when the city of New York launched a massive aerial spraying of it. Shown to be mutagenic, a possible carcinogen, implicated in vision loss, reproductive and learning problems, immune system disruption and other negative health effects in human and animal studies, damaging to non target organisms, and containing highly toxic impurities, Malathion has a legacy of serious problems. Recent evidence suggests that organophosphates such as Malathion can cause Non-Hodgkin's Lymphoma (NHL). During a malaria mosquito eradication spray program in Pakistan in 1976, 2,800 people became poisoned from Malathion and 5 died. Physicians at Travis Air Force Base Medical Center in California have observed seven children with bone marrow disorders over the past 8 years. The physicians believe organophosphate pesticides caused the blood disorders, in all cases. All blood disorders occurred shortly after exposure to the pesticides DDVP/propoxur and Malathion. It is capable of inhibiting carboxyesterase enzymes in those exposed to it.

Malathion is lethal to beneficial insects, snails, micro crustaceans, fish, birds, amphibians, and soil microorganisms. Sublethal exposure of these species can cause a variety of behavioral and physiological abnormalities. Malathion undergoes a chemical reaction in sunlight called "photolysis" which results in increasing the formation of the highly toxic trimethyl impurities. In studies of the effects of long-term exposure to oral ingestion of malaoxon in rats, malaoxon has been shown to be 61 times more toxic than Malathion. The organophosphorus insectides (malathion and Diazinon) in the area of kajraj river with high performance thin layer chromatography (HPLC) were determined and the environment are at risk of chronic toxicity with Malathion and Diazinon through consuming polluted water and agricultural products [2]. Raafat et al. (2012) [3] suggested that chronic exposure of non-diabetic farmers to organophosphorus Malathion pesticides might induce insulin resistance. This effect tended to strengthen as waist circumference increases. An increased health and environmental hazard may occur in areas with intensive agricultural production. The environmental consequences of delayed effects and embryotoxicity for bird populations in areas exposed to organophosphate insecticides, such as Malathion, are obvious [4]. Yonar (2013) [5] demonstrated that the exposure of carp to Malathion resulted in alterations in the haematological profiles and immune responses, and lead to increased reactive oxygen species formation, resulting in oxidative damage and inhibition of the antioxidant capacities. However, the administration of lycopene prevented malathion-induced toxic effects.

Increased and persistent use of pesticides since 1994, to boost crop productivity has resulted in the entry of large amounts of pesticides into various ecosystems with actually only a small portion reaching the targeted sites. Today pesticides are extensively used not only in agriculture but also for many diverse purposes such as human and animal health protection, pest control, in forest and aquatic environments and protection of buildings and other structures. Contamination of surface waters and ground water by pesticides is a major environmental concern. Organophosphorus pesticides like Malathion are among the most widely used pesticides in non-crop areas as well as in food crops. Given the potential carcinogenic risk from the pesticides there is serious need to develop remediation processes to eliminate or minimize contamination in the environment. Biodegradation could be a reliable and cost effective technique for pesticide abatement.

The transformation of pesticides in the environment results from physicochemical reactions as well as from the activity of cellular or extracellular components of the biota (microorganisms, plants, and animals), but the principal biological pathway is microbial degradation: microorganisms can metabolize various pesticides both in soil and in water. The earlier metabolic studies on pesticides helped to develop a new approach to the detoxification of pesticides using cell-free enzymes from adapted microorganisms to resolve problems related to whole-cell metabolism of pesticides. Since today as there were no metabolic pathway predicted for the degradation of organophosphates pesticide Malathion in KEGG database or in any of the other pathway databases. Thus in the present study, an attempt has been made to predict the microbial biodegradation pathway of Malathion using bioinformatics tools through the following steps, retrieval of structure of the Malathion, retrieving the Smile form of the Malathion and pathway prediction of Malathion.

## Methodology

### Structure Retrieval

PubChem Structure Search is a chemical structure similarity search tool that links to the PubChem Compound and PubChem Substance databases. Searches may be specified using SMILES, MOL file, or molecular formula. The structure of organophosphorus insecticide Malathion was obtained from Pubchem Structure search and the website utilized was http://pubchem.ncbi.nlm.nih.gov/search.

### SMILES Retrival

The simplified molecular input line entry specification or SMILES is specification for unambiguously describing the structure of chemical molecules using short ASCII strings. SMILES strings can be imported by most molecule editors for conversion back into 2- dimensional drawings or 3-dimensional models of the molecules. The canonical smile form of Malathion was obtained from pubchem structure search. The website was http://pubchem.ncbi.nlm.nih.gov/search/. The retrieved smiles is as follows:

CCOC(=O)CC(C(=O)OCC)SP(=S)(OC)OC

### Pathway Prediction

The eawag-bbd pathway prediction system predicts microbial catabolic reactions using substructure searching, a rule-base, and atom-to-atom mapping. The system is able to recognize organic functional groups found in a compound and predicts transformations based on biotransformation rules. The biotransformation rules are based on reactions formed in the eawag-bbd database. Rules are based on reactions found in the Marvin sketch and Marvin view java applets as plugins. The website was http://eawag-bbd.ethz.ch/predict/ [1].

## Result and discussion

The canonical smile of Malathion was obtained from pubchem structure search and the biodegradation pathway of Malathion was predicted using EAWAG-BBD pathway prediction system. Malathion is degraded to its monoacid and diacid derivatives through carboxyl esterase activity. Several other studies also suggested that it is the predominant metabolic mechanism [6-9]. Most of the microorganisms’ posses the carboxyl esterase activity is shown in Table 1.

For several microorganisms Malathion serves as an essential sole carbon source, which metabolizes Malathion for its survival, Horne et al. (2002) [10] found that Agrobacterium radiobacter has the ability to hydrolyze a wide range of organophosphate insecticides. Further, a wide range of parathion degrading bacterial strains was isolated and identified as Pleisomonas sp. Roy et al. (1995) [11] isolated five Malathion degrading bacterial strains, identified as pseudomonas and micrococcus from soil during their search for facultative anaerobes capable of degrading Malathion. [12-14] Several authors found that some soil microorganisms especially Streptomyces sp., pseudomonas sp., Streptomyces rimosus, Fusarium moniliform can utilize Malathion and other organophosphorus pesticides under in-vitro conditions. Matsumara & Boush (1968) [6] found that certain Trichoderma viridae had very marked ability to breakdown Malathion through the action of carboxyl esterase. Walker & Stojanovic (1974) [7] noticed that Athrobacter sp. was able to degrade Malathion to Malathion mono and dicarboxylic acids.

Muan & Skare (1989) [15] found that biodegradation of Malathion to inactive metabolites results from hydrolytic cleavage of one or two of the carboxyl groups giving rise to mono-or-di-acids respectively. Malathion degradation by fungal cutinase and yeast esterase to MMA and MDA were reported [16]. Apart from these enzymes, the pathway was also involved with several enzymes such as Oxidases and Oxidoreductases activity in the biodegradation of Malathion. Several microorganisms’ posses these enzymatic activities are tabulated in Table 2 and Table 3 respectively which helps to predict the degradation pathway. Bourquin (1977) [8] isolated 11 bacterial isolates from salt marsh environments after Malathion enrichment, which utilized Malathion as a sole carbon source. Some fungi including Aspergillus niger and Penicillium notatum, and Rhizobium legiminosarum are involved in the degradation of malathion.

Finally small amounts of other metabolites were also produced including desmethyl Malathion, phosphorodithoates and four carbon dicarboxylic acids, which were probably formed as a result of phosphatase activity. Some of the Rhizobium sp. finally produces inorganic phosphate and finally it is degraded into methanol. Some of the microorganisms involving phosphatase activity were shown in the Table 4. The methanol is then converted into formaldehyde and finally forms carbon dioxide and hydrogen, which is non-toxic to the environment. A gene encoding a protein involved in organophosphorus pesticides hydrolysis (opd gene) was cloned from Agrobacterium radiobacter p230 and sequenced [10]. Acinetobacter and Bacillus spp. Bacterial strains could degrade more than 90% of the initial Malathion concentration (1000 ppm) within 4 days [17].

## Conclusion

Malathion is a common pesticide used to control insects in agricultural, domestic and industrial sectors in different parts of the world [18]. The structure and its SMILE form of malathion were obtained and predicted the pathway using EAWAG-BBD pathway prediction system, which involves a series of reactions resulting in the biodegradation finally leading to the conversion of malathion to a non-toxic compound such as dimethyl sulfanyl phosphonate which can be degraded easily as carbon dioxide and hydrogen with the help of common pathway which is available in most of the microbes.

The pathway involves enzymes such as carboxyl esterase, Oxidases, Oxidoreductases, phosphatases and dehydrogenase which is present in numerous number of soil, water and airborne microorganisms of which some of them posses all enzymatic properties and that particular microorganism is enough to degrade the toxic organophosphates such as malathionacetylcholine inhibitors. Evidences report that some of the Streptomyces sp. (Streptomyces griseus) is able to degrade the toxic compound such as malathion but it lacks some of the enzymes required for degradation and this may be overcome by Escherichia coli as it posses all the enzymes for degrading Malathion.

By evaluating the culture characteristics, nature of the organisms Escherichia coli considered to be the most effective microbe in the degradation of Malathion. Economical wise also it is very convenient, as it will actively grow on easily available medium such as Eosin Methylene Blue (EMB) or Mac Conkey Agar for various economical purposes, it is reported that most of the strains of E. coli are non-pathogenic so that it can be easily cultured and used without any harm. The present study result is only from in-silico prediction but it might be proved with the help of wet-lab studies. The pathway prediction tools are gaining momentum, since this approach is proven to be fruitful in several biodegradation studies and reduced considerable money and time spent on wet lab research.

## Figures and Tables

**Table 1:** Microorganisms Containing Carboxyl Esterase Enzyme

Enzyme	Organism	Gene ID
I	*Bacillus subtilis subsp. subtilis str. 168 *	AL009126.3
	*Saccharomyces cerevisiae *	FN394216.1
	*Capnocytophaga ochracea DSM 7271*	CP001632.1
	*Flavobacterium johnsoniae UW101*	CP000685.1
	*Maribacter sp. E4-6 *	FN377745.1
	*Zobellia sp. BSs20189 *	DQ514305.1
	*Pibocella ponti isolate S3-17 *	AY771726.1
	*Salmonella enterica*	NC_00319197.1
	*Klebseilla pneumoniae*	NC_009648.1
	*Aeromonas hydrophila*	NC_008570.1
	*Streptococcus mutans UA159*	NC_004350.1
	*Clostridium perfringens strain-13*	NC_003366.1
	*Aspergillus niger super contig An11*	AM270990.1
	*Caenorhabditis elegans *	Z78416.1
	*Chlamydomonas reinhardtii*	XM_001702395
	*Actinomyces odontolyticus ATCC17982*	NZ_DS264586.1
	*Staphylococcus aureus A9781 cont 1.37*	NZ_ACKL01000037.1
	*Arthrobacter sp.*	NC_008541.1
	*Escherichia coli SMS-3-5*	NC_010498.1
	*Penicillium chrosogenum Wisconsin 54.1255 *	NS_000201.1
	*Yersinia pestis KIM 10*	NC_004088
	*Agrobacterium radiobacter K84*	NC_011983.1

**Table 2:** Microorganisms Containing Oxidase Enzyme

Enzyme	Organism	Gene ID
II	*Leuconostoc kimchii IMSNU11154*	CP001758.1
	*Pediococcus pentosaceus ATCC 25745*	CP000422.1
	*Enterococcus dispar *	AJ295303.1
	*Listeria seeligeri serovar 1/2b str. SLCC3954 *	FN557490.1
	*Streptococcus pyogenes MGAS10750*	CP000262.1
	*Clostridium difficile strain M120*	FN665653.1
	*Anaerococcus prevotii DSM 20548 *	CP001709.1
	*Sanguibacter keddieii DSM 10542*	CP001819.1
	*Micrococcus luteus NCTC 2665*	CP001628.1
	*Brachybacterium faecium DSM 4810 strain 6-10*	CP001643.1
	*Kineococcus radiotolerans SRS30216*	CP000750.2
	*Thermobifida fusca YX*	CP000088.1
	*Rothia mucilaginosa DY-18 *	AP011540.1
	*Catenulispora acidiphila DSM 44928*	CP001700.1
	*Amycolatopsis lurida *	X81576.1
	*Streptomyces griseus subsp. griseus NBRC 13350 *	AP009493.1
	*Kocuria rhizophila DC2201 *	AP009152.1
	*Geodermatophilus obscurus DSM 43160*	CP001867.1
	*Actinosynnema mirum DSM 43827*	CP001630.1
	*Staphylococcus aureus *	NZ_ACJA02000004.1
	*Beutenbergia cavernae DSM 12333*	CP001618.1
	*Xylanimonas cellulosilytica DSM 15894*	CP001821.1
	*Escherichia coli SMS-3-5*	NC_010498.1
	*Brucella abortus*	NZ_GG774528.1

**Table 3:** Microorganisms Containing Oxidoreductases Enzyme

Enzyme	Organism	Gene ID
III	*Vibrio cholerae MJ-1236 *	CP001485.1
	*Photobacterium damselae subsp. piscicida*	AJ870986.2
	*Providencia alcalifaciens *	GQ463139.1
	*Escherichia coli SMS-3-5*	NC_010498.1
	*Proteus mirabilis*	AB525688.1
	*Aspergillus niger *	AM270075.1
	*Yersinia pestis*	NC_010159.1
	*Salmonella enterica strain AM04528*	FJ621587.1
	*Anaerococcus prevotii DSM 20548*	CP001709.1
	*Streptomyces griseus subsp. griseus NBRC 13350 *	AP009493.1
	*Streptococcus agalactiae A909*	CP000114.1
	*Mycobacterium tuberculosis*	NC_000962.2
	*Bacillus cereus*	AE016877.1
	*Staphylococcus aureus*	NZ_ACJA02000004.1
	*Brucella abortus*	NZ_GG774528.1

**Table 4:** Microorganisms Containing Phosphatase Enzyme

Enzyme	Organism	Gene ID
IV	*Acinetobacter baumannii AB307-0294*	CP001172.1
	*Enterobacter hormaechei 05-545*	FN297818.1
	*Citrobacter koseri ATCC BAA-895*	CP000822.1
	*Vibrio sp. Ex25 *	CP001805.1
	*Alteromonas macleodii 'Deep ecotype'*	CP001103.1
	*Bacteroides vulgatus ATCC 8482*	CP000139.1
	*Vibrio vulnificus CMCP6*	AE016795.2
	*Callorhinchus milii *	AC239486.2
	*Bacillus subtilis subsp. subtilis str. 168 *	AL009126.3
	*Clostridium difficile BI9 *	FN668944.1
	*C.sordelii cytotoxin *	X82638.1
	*Faecalibacterium prausnitzii L2/6 *	FP929045.1
	*Enterococcus faecium strain D344R*	DQ321786.1
	*Streptococcus equi subsp. zooepidemicus MGCS10565*	CP001129.1
	*Corynebacterium glutamicum ATCC 13032 *	BA000036.3
	*Streptomyces griseus subsp. griseus NBRC 13350 *	AP009493.1
	*Photorhabdus asymbiotica ATCC43949 complete genome*	FM162591.1
	*Shewanella woodyi ATCC 51908*	CP000961.1
	*Escherichia coli SMS-3-5 *	NC_010498.1
	*Staphylococcus aureus*	NZ_ACZQ01000078.1

**Figure 1:**
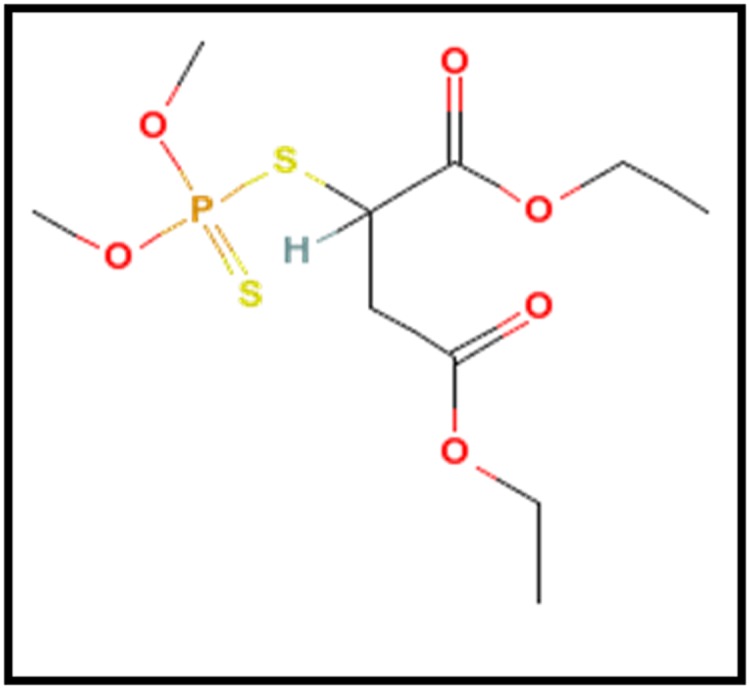
Structure of Malathion

**Figure 2:**
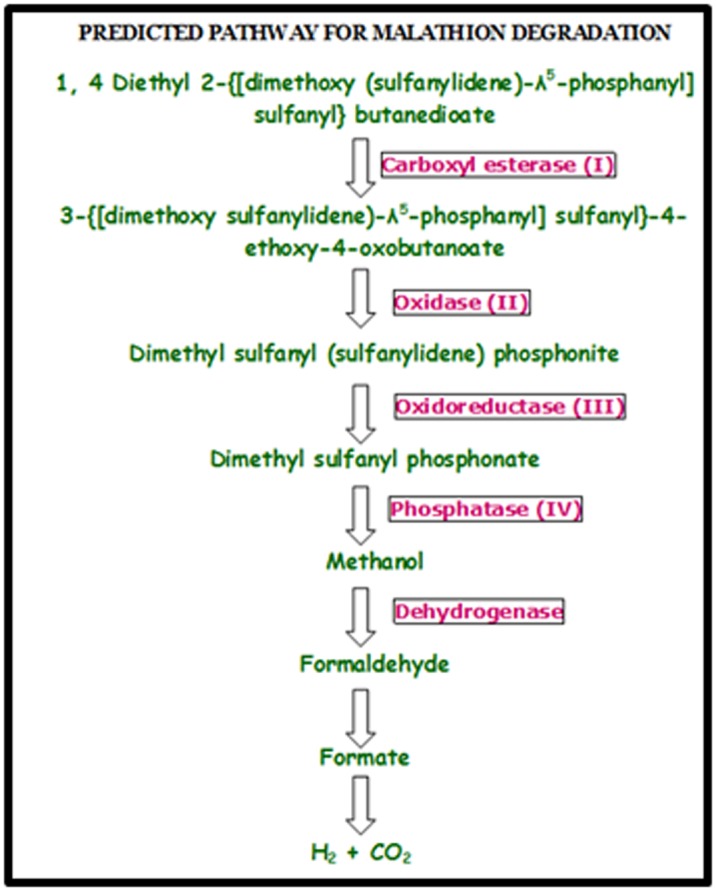
Predicted pathway

**Figure 3:**
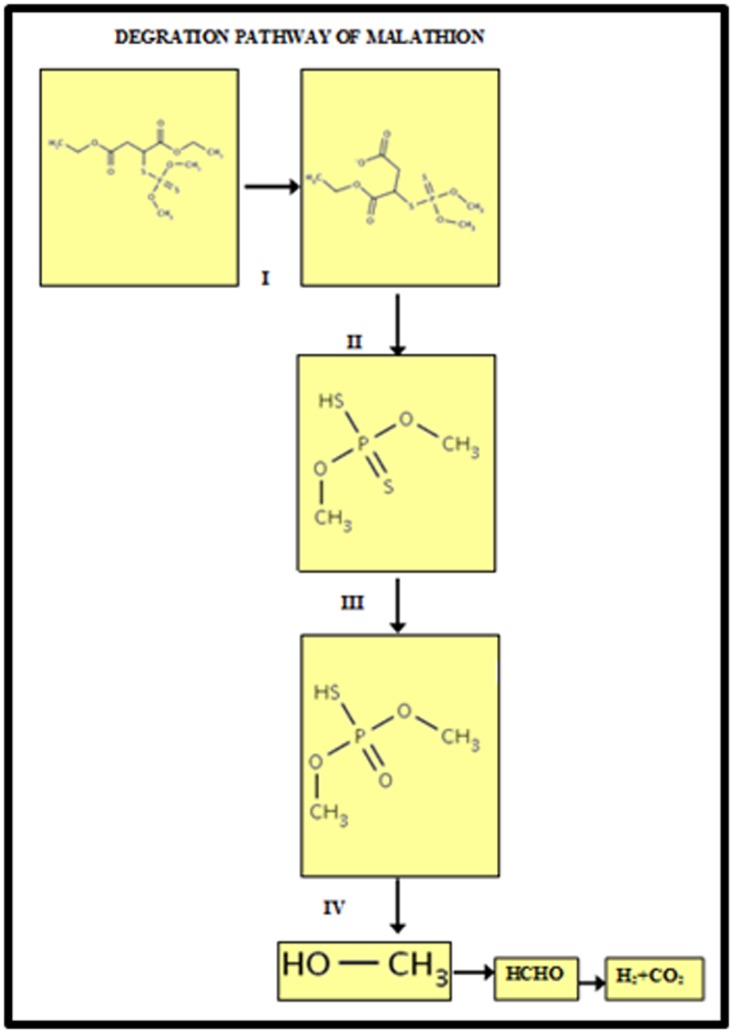
Predicted degradation pathway with structures
